# An optimization framework for measuring spatial access over healthcare networks

**DOI:** 10.1186/s12913-015-0919-8

**Published:** 2015-07-17

**Authors:** Zihao Li, Nicoleta Serban, Julie L. Swann

**Affiliations:** H. Milton Stewart School of Industrial and Systems Engineering, Georgia Institute of Technology, Atlanta, USA; School of Public Policy by courtesy, Georgia Institute of Technology, Atlanta, USA

**Keywords:** Optimization, Health access, Measurement of access, Intervention

## Abstract

**Background:**

Measurement of healthcare spatial access over a network involves accounting for demand, supply, and network structure. Popular approaches are based on floating catchment areas; however the methods can overestimate demand over the network and fail to capture cascading effects across the system.

**Methods:**

Optimization is presented as a framework to measure spatial access. Questions related to when and why optimization should be used are addressed. The accuracy of the optimization models compared to the two-step floating catchment area method and its variations is analytically demonstrated, and a case study of specialty care for Cystic Fibrosis over the continental United States is used to compare these approaches.

**Results:**

The optimization models capture a patient’s experience rather than their opportunities and avoid overestimating patient demand. They can also capture system effects due to change based on congestion. Furthermore, the optimization models provide more elements of access than traditional catchment methods.

**Conclusions:**

Optimization models can incorporate user choice and other variations, and they can be useful towards targeting interventions to improve access. They can be easily adapted to measure access for different types of patients, over different provider types, or with capacity constraints in the network. Moreover, optimization models allow differences in access in rural and urban areas.

**Electronic supplementary material:**

The online version of this article (doi:10.1186/s12913-015-0919-8) contains supplementary material, which is available to authorized users.

## Background

Access to healthcare is widely recognized as essential for ensuring not only care of immediate health needs but also to enable health and wellness in the population. Access has multiple dimensions including accessibility, availability, affordability, accommodation, and acceptability [1–3] and is of great importance to decision makers in public health. In this paper, we focus on measurement models for *spatial* access over a health network with patients and providers, which is most closely related to the elements of *accessibility* (e.g., location and travel distance for care) and *availability* (e.g., coverage or the volume of providers). A healthcare network is defined as a transportation network with patients as demand nodes and providers as supply nodes, and an arc between patient and provider if the provider is accessible for the patient.

The measurement models studied in this paper are designed to measure *potential* access based on the services that are available for use relative to population and distance. On the contrary, *realized* access reflects actual use of services, which can be affected by finances, behaviors, and other factors. Potential access is measurable although it is not observable.

An optimization-based approach is described in this paper for quantifying potential access over the healthcare network and for estimating the impact of changes to the network. Optimization is a mathematical science that is widely accepted in engineering and science as providing a way to balance complex interactions across a system, and there is a history of using optimization to assist medical decision making [4–6]. In this paper, theoretical and practical optimization modeling techniques are used to assist with health care policy development by measuring access and computing the economics behind discrepancy of access. Specifically, questions such as how optimization models can be used to measure access, on what types of networks they offer the most accurate estimates of access, and ultimately, why they should be used for measuring and for suggesting interventions to improve access are addressed in this paper. The answers to these questions are useful for improving the health of populations and assisting with health policy development by informing areas of greatest need.

The optimization models are compared to some existing methods. In particular, comparisons are made to variations of the two-step floating catchment area (2SFCA) method [7], including the Enhanced 2SFCA (E2SFCA) method [8] and the Modified 2SFCA (MSFCA) method [9], with some discussion of other catchment methods. The catchment methods, which are offsprings of a gravity model of attractions between populations and providers, estimate the size of population served at each provider using distance zones and compute accessibility of a community based on the availability of providers in the community’s zones; communities can be captured in the zones of multiple providers. In contrast, optimization models match patients and providers based on both distances and the relatively crowdedness of each provider, and estimate the accessibility of a patient using the matching results to determine the travel distance and the corresponding crowdedness of each patient. Optimization models can take on the perspective of a centralized planner in making assignments, or they can be adapted to directly incorporate patient choice over the network.

To compare the measurement models for spatial access, several specific network structures are examined, which are designed so that access measures can be compared analytically. Results on a large case study of specialty care of Cystic Fibrosis (CF), where the network has varying levels of accessibility are also provided.

Analytically, this paper demonstrates that the total number of patient visits captured by all facilities in the 2SFCA methods is larger than the number of visits expected based on population size. The three-step floating catchment area (3SFCA) method [10] adds an assignment mechanism to address the competition by facilities, but the assignments are only based on distance. In contrast, in the optimization models, the willingness to travel is not only a function of distance but also of facility congestion including its size. As a result, the optimization models can capture cascading effects in the system, where a change in congestion for one population leads to different decisions and thus impacts individuals in another location. The optimization models also allow for simultaneous estimation of measures of access across the five dimensions outlined [1].

More generally, optimization models can be adapted to many contexts including different patient types (e.g., Medicaid or not), provider constraints, or others. They are also useful in optimizing interventions, where the intervention can target different aspects of access (e.g., distance versus congestion).

## Methods

### Optimization framework

In healthcare decision making and service research areas, optimization models have been used to determine the best location for a new clinic [11–13], ensure that resource locations are sufficient to cover the need across a network [14], route nurses for home health services [15], improve health outcomes among communities [16, 17], and evaluate policies for pandemic influenza, breast cancer, and HIV over a network [18–20], among others. Wang [21] reviewed several cases where optimization models could be used to improve access or service over a network.

In our models, the cost of an individual is associated with two dimensions of access [1]: accessibility and availability. The first is measured with travel distance (or time). The second is measured with congestion, which for an individual is associated with the relative number of people (or visits) at a provider compared to the resources available. One can also think of this as capturing the waiting time until an appointment is available. Studies show that individuals are willing to drive further to receive an appointment more quickly [22]. Thus we assume that the utility (or disutility) associated with a patient’s access is a weighted sum of the distance and a congestion term, where we scale the congestion term to trade-off the relative importance between the two. We expect that the congestion weight (α) may be different for different types of healthcare services, such as primary care or specialty care (i.e., distance may have a relatively lower cost). The congestion weight can also represent the resources available at a facility.

Several elements are defined for our formulation. The total number of patients is *n* and the total number of facilities is *m*. Let *i* = 1,…, *n* be the indices of patients and *j* = 1,…, *m* be the indices of facilities. The distance between patient *i* and provider *j* is *d*_*ij*_; *v*_*i*_ is the estimated number of visits that patient *i* = 1,…, *n* will make (demand);and α_*j*_ is the congestion weight at provider *j*. A dummy location can be introduced for the assignment of demand that cannot be met.

The decision variables are *x*_*ij*_, which is the percentage of time assigned to facility *j* from patient or community *i*, for each *i* = 1,…, *n* and *j* = 1,…, *m*.

The formulation of the basic centralized model follows:

*Objective function:*

1 $$ \min {\displaystyle {\sum}_{i=1}^n{\displaystyle {\sum}_{j=1}^m{d}_{ij}{x}_{ij}{v}_i}}+{\displaystyle {\sum}_{j=1}^m{\alpha}_j{\left({\displaystyle {\sum}_{i=1}^n{x}_{ij}{v}_i}\right)}^2} $$

*Constraints:*

2 $$ {\displaystyle {\sum}_{j=1}^m{x}_{ij}{v}_i={v}_i,\forall i=1,\dots, n}\kern1.68em \left(\mathrm{assignment}\ \mathrm{constraint}\right) $$

3 $$ 0\le {x}_{ij}\le 1,\forall i=1,\dots, n\;\mathrm{and}\forall j=1,\dots, m. $$

The objective function (1) states that the total number of visits assigned should be *v*_*i*_ for each patient or community *i*. Constraint (2) requires that all individuals be assigned, and equation (3) requires non-negativity of the decision variables. Each individual’s congestion at a visit is proportional to the total number of visits at that facility scaled by α_*j*_. The congestion term in the objective sums over the congestion experienced by all patients resulting in an overall term that is squared. The choice of quadratic function comes from the following idea: if *n* patients receive care from a provider location, then each patient experiences *n* units of congestion, then the total congestion is *n* × *n = n*^*2*^ (similar to total latency in network congestion work [23]). Note that when α = 0, this model gives equivalent results to assignment by shortest distance, and when α = ∞, this model gives equivalent results to equally distributing patient visits to each facility. See Additional file 1 section 1 for a process to select the congestion weight.

For a patient, the number of visits to a close location is expected to be more than the number of visits to a far location because of the willingness to travel. Thus, the number of visits to each location using a function that decays with distance is determined. This is analogous to step 1 in the E2SFCA method where the population is multiplied by a weight. This also implies that the number of visits covered in the network may be less than 100 %.

From the results of an optimization model, several measures of spatial access are calculated. The measures include i) the distance traveled for each patient or community; ii) the congestion experienced by each patient or community; iii) the coverage, which is defined as the ratio of visits assigned to visits needed for an individual or community.

### Variations on the optimization model

With optimization models, many variations are possible, including through the addition of constraints, the use of different objective function values, or by differentiating decision variables by type. Here we describe a major variation in our model, *optimization with user choice (“Decentralized”)*, and include many others such as capacity, unmet demand, and willingness to travel in Additional file 1 section 2.

The traditional deterministic optimization model (as presented above) often assumes a centralized planner who makes decisions for every patient in a healthcare network to achieve the best overall objective. However, user choice can be incorporated by an equilibrium constraint that represents individual choices as in game theory [24]; we call the resulting optimization model *decentralized*.

An overall equilibrium solution requires a user choice constraint to be satisfied for each patient visit in the network, where the constraint states that the individual cannot improve their distance and congestion of that visit by switching to another facility given the other decisions on the network.

The decision variable and equilibrium constraint are defined below:

*x*_*ijk*_ = decision variable is 1 if patient *i* chooses facility *j* for visit *k*, or 0 otherwise;

4 $$ {d}_{ij}+{\alpha}_j{\displaystyle {\sum}_{p=1}^n{\displaystyle {\sum}_{k=1}^{v_p}{x}_{pjk}\le {d}_{iq}+{\alpha}_q}\left({\displaystyle {\sum}_{p=1}^n{\displaystyle {\sum}_{k=1}^{v_p}{x}_{pqk}+1}}\right),\forall q\ne j,\forall i,\forall k} $$

The equilibrium condition includes a separate constraint for each patient’s visit and each location when there is no distance decay function. The left-hand side is the distance and congestion associated with current facility choice *j* for a visit *k*, and the right-hand side is the distance and congestion at any location other than *j*. See Additional file 1 section 3 for more details.

### Review of catchment models

Gravity models use the following general form to calculate an “attraction” measure for each patient *i*:

5 $$ {A}_i^G={\displaystyle {\sum}_{j=1}^m\frac{S_jw\left({d}_{ij}\right)}{{\displaystyle {\sum}_{i=1}^k}{P}_iw\left({d}_{ij}\right)}}, $$

where *S*_*j*_ is the supply at provider *j*, *P*_*i*_ is the population at location *i, w*(*d*_*ij*_) is the decay function based on distance of each patient-provider pair (*i*,*j*).

The original 2SFCA method was introduced by Luo and Wang [7]; it allows the catchment of each provider and patient to float based on the distances between each pair. E2SFCA is a variation that suggests applying different weights within travel time zones to account for decaying of the willingness to travel as distance increases [8]. Under the E2SFCA model, in the first step the “physician-to-population ratio” at each provider is calculated. Although the E2SFCA aims to estimate the number of *patients* that may potentially use a facility, it is easy to extend the metrics to estimate the number of *visits* by replicating each patient using visits demanded (e.g., a patient demanding 10 visits can be viewed as 10 patients) [25, 26]. We make a minor adjustment to allow for each patient to have multiple visits to a provider, so we use physician-to-visits ratio instead. Thus we obtain:

6 $$ {R}_j=\frac{S_j}{{\displaystyle {\sum}_r}{\displaystyle {\sum}_{i\in \left\{{d}_{ij}<{D}_r\right\}}}\;{V}_i{W}_r}, $$

where *S*_*j*_ is the number of physicians available at provider *j*, *W*_*r*_ is the weight value corresponding to the catchment zone of *d*_*ij*_. The value of *W*_*r*_ is calculated using the distance decay function, which is usually nonlinear. *D*_*r*_ is the distance threshold of catchment zone *r*. The parameter *V*_*i*_ is the number of potential visits if there is no decay in willingness to travel or the maximal demand for patient or community *i*. The original E2SFCA method introduced the model with three catchment zones, but an extension is to allow a different number of zones or even a continuous decay (“impedance”) function across a single zone. Example choices of impedance functions include Gaussian [7, 27], exponential, inverse power, and others; [27] discusses parameter setting for the impedance function. In the second step of E2SFCA, the method defines the accessibility of each patient or community *i* based on the ratios at each provider and the zone weights:

7 $$ {A}_i = {\displaystyle {\sum}_r{\displaystyle {\sum}_{j\in \left\{{d}_{ij}<{D}_r\right\}}{R}_j{W}_r.}} $$

Another catchment approach is the 3SFCA method, which incorporates competitions among multiple providers within the same catchment zone of a patient and makes assignments of patients by distance. The M2SFCA method [9] modifies the patient level accessibility in [7] by multiplying the distance weight twice, while another approach [28] allows for zones to differ by transportation modes.

For a simple system, the individual measures of spatial access from optimization models can be combined to directly compare with the accessibility measures of 2SFCA methods (E2SFCA and M2SFCA). The simplest supply network consists of n communities in a circular population area with a facility at the center. Let *d*_*i*_ be the distance from community *i* to the facility and *S* the number of physicians in the facility. Calculate the facility population-to-physician ratio *R* and patient accessibility*A*_*i*_ using [6] and [7]. Define a decay function *w* (*d*_*i*_) ∈ [0,1]*.* For this system, the optimization method is equivalent to assigning by shortest distance. Let *F* denote the congestion at the facility, then $F=\frac{1}{R}$. The coverage of community *i* is calculated as w(*d*_*i*_). Therefore, for this system, the patient accessibility is ${A}_i^E=\frac{coverage}{congestion}$, for the E2SFCA method. For the M2SFCA method, a similar calculation can be made, where the composite patient accessibility measure is ${A}_i^M=\frac{coverag{e}^2}{congestion}$.

### Human subject study approval

The Institutional Review Board of the Georgia Institute of Technology approved the overall research project using data from the Cystic Fibrosis Foundation, and the Cystic Fibrosis Foundation also approved the study to use registry data previously collected from patients with their signed consent. The submitted article uses the existing locations of Cystic Fibrosis care centers, the distances traveled by patients to CF centers for care, and simulated patient locations with corresponding distances to CF centers. Simulated locations of patients are randomly generated according to the prevalence of CF and the composition of populations at the county level.

## Results

### Analytical comparisons

In this section, analytical results on accessibility as measured by the optimization method and catchment models are provided. Most analyses in this section focus on simple systems where service areas are non-overlapping. For simple networks with overlapping service areas, the detailed analysis can be found in Additional file 1 section 4. Notations that will be used frequently in the analysis are defined below. The distance decay function *w*(*d*_*ij*_) is between 0 and 1. If *d*_*ij*_ is the distance between community *i* and facility *j*, and *v*_*i*_ is the visits needed by community *i*, then we assume that facility *j* receives *w*(*d*_*ij*_)*v*_*i*_ visits from community *i* as in the catchment models*.* In optimization models, let *P*_*ij*_ be the proportion of the population in community *i* that visits facility *j.*

#### Result 1 (Opportunities vs. Experiences): optimization models capture a patient’s experience rather than their opportunities. As a result, 2SFCA methods tend to overestimate the total number of visits

For many catchment models, the estimated accessibility measure increases when more facility choices are available to a population. However, assignments models (including optimization and the 3SFCA method), are estimating the cost of potential access, and this does not increase if a new choice is congested or inconsequential. This is illustrated with a simulated system of populations and facilities, as in Delamater (2013) [9] .

**Fig. 1:**
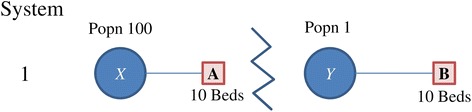
System 1, with populations 100 at location *X* and 1 at *Y*. Facilities (**a**) and (**b**) each have 10 beds

Consider System 1 as described in Fig. 1. When facility *A* and population *X* are sufficiently far from *B* and *Y*, the catchment models and the optimization method will provide the same accessibility estimate. Consider a second system, where *B* and *Y* are both closer to *X* and *A* than in the first system, with the distances between *A - X* and *B - Y* retained and b closer to *Y* than *A*. The 2SFCA methods show that the accessibility of *Y* increases due to the possibility of service at *A*, while the accessibility of *X* decreases because of demand on facility *A* from population *Y*. However, the optimization method shows there is no change in accessibility for reasonable congestion weights. From the perspective of a person at *Y*, service at facility *A* would be associated with a higher congestion cost and a further distance, thus he would neither be assigned to facility *A* nor choose that facility. This is still the cost associated with *potential* access rather than realized access, but the cost is associated with the potential *experience* of a patient. In contrast, the 2SFCA methods always realize additional choices regardless of their relative competitiveness to existing choices. Therefore the total number of visits implied by the 2SFCA methods is higher compared to the optimization method, and can be higher than the total number of visits demanded.

**Fig. 2:**
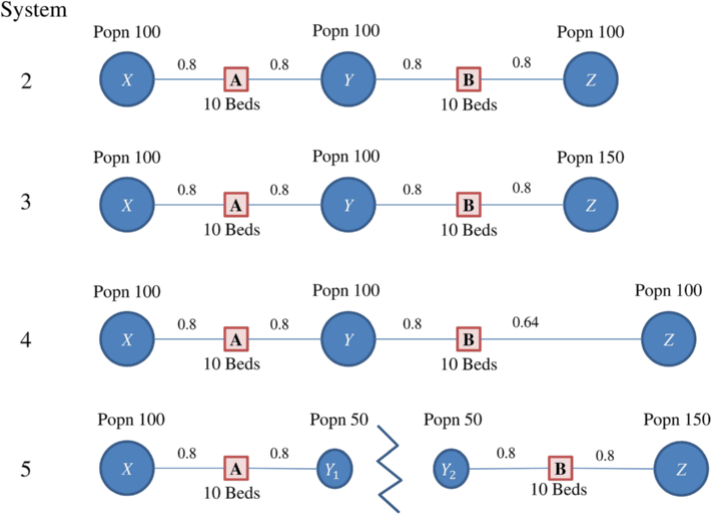
Systems 2 through 5, with populations as specified at location *X, Y*, and *Z*. Facilities (**a**) and (**b**) each have 10 beds, and the distance weights are provided between locations

**Fig. 3:**
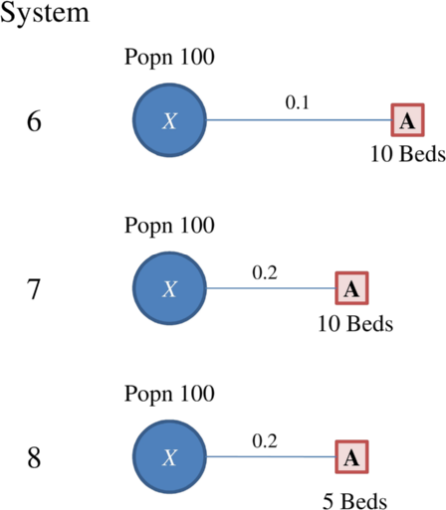
Systems 6 ~ 8, with population of 100 at location *X*, and a single facility with either 5 or 10 beds. Distance weights are provided for each system

#### Result 2 (System Effects): the 2SFCA methods do not capture the cascading effects based on congestion

For methods focused primarily on catchment zones without assignment, there are some system effects that may not be captured over the network. In Fig. 2, we define several systems to illustrate this point. Define System 2, with population *z* added to system 1, and with a population of 100 for each of *X*, *Y*, and *Z*. In this system, the optimization method and the 3SFCA both compute the same accessibility for each population, while in the 2SFCA methods the accessibility is higher for *Y* since it is capturing opportunities for access rather than the patient experience.

Consider System 3 with increased population at location *Z*. In the catchment models, as the population of *Z* increases, the accessibility for *Y* and *Z* decrease, while the accessibility for *X* remains the same no matter how large *Z* is. In the optimization method, as *Z* gets larger, more of the population from *Y* goes to facility *A*, so the accessibility at all population locations decreases. The accessibility at each location is the same because the system is constructed in a very specific and symmetric way.

A similar effect can be seen when System 2 is varied by moving population *Z* further away from the center (System 4). In this case, more patients from *Y* switch to B to reduce congestion, resulting in better access for population *X* in the optimization method, while the 2SFCA methods show no change for *X*.

Define System 5 the same as 1 but with an unbreakable barrier separating population *Y* in half, and a population of *Z* equal to 150. The 3SFCA quantifies the same access with and without the barrier, because the assignment is based on distance alone. On the other hand, the optimization method shows different access in System 5 compared to 3, because assignment is based on both distance and congestion. The accessibility estimates for the different systems are summarized in Table 1.

**Table 1:** Accessibility estimates for systems 2 ~ 5

	E2SFCA			M2SFCA		
System	X	Y	Z	X	Y	Z
2	0.05	0.1	0.05	0.04	0.08	0.04
3	0.05	0.0833	0.0333	0.04	0.0667	0.0267
4	0.05	0.1056	0.0444	0.04	0.0844	0.0284
5	0.067	Y_1_ = 0.067 Y_2_ = 0.05	0.05	0.053	Y_1_ = 0.053 Y_2_ = 0.04	0.04
	Optimization (A^E^)			Optimization (A^M^)		
System	X	Y	Z	X	Y	Z
2	0.067	0.067	0.067	0.053	0.053	0.053
3	0.057	0.057	0.057	0.046	0.046	0.046
4	0.071	0.071	0.0571	0.0571	0.0571	0.0366
5	0.067	Y_1_ = 0.067 Y_2_ = 0.05	0.05	0.053	Y_1_ = 0.053 Y_2_ = 0.04	0.04

#### Result 3 (Composite Measures vs. Individual Measures): the composite measures of the 2SFCA methods are insufficient to distinguish multiple elements of access

Consider systems 6 ~ 8 in Fig. 3. System 6 has 100 people in *X* and 10 beds in *A*, and the distance weight between *X* and *A* is 0.1. System 7 is similar to system 6 but with a distance weight 0.2 (which implies the population is closer to the facility). System 8 is similar to system 7 but has 5 beds in *A.* As we move from system 6 to system 7 and then to system 8, either the population is closer to the facility, the facility has fewer beds, or both, so the network is getting more congested and the accessibility of *X* should reflect this change. However, as Delamater [9] points out, the E2SFCA method shows the same accessibility for populations in system 6 and 7. Similarly, the M2SFCA method shows the same accessibility for populations in system 6 and 8.

The individual measures in the optimization method indicate the coverage increases as you move to system 8 but that the congestion also increases (see Table 2).

**Table 2:** Accessibility estimates for systems 6 ~ 8

System	E2SFCA	M2SFCA	Opt coverage	Opt congestion
6	0.1	0.01	0.1	1
7	0.1	0.04	0.2	2
8	0.05	0.01	0.2	4

### Case study

The analytical analysis above illustrates several direct comparisons between the 2SFCA methods and the optimization method. In this section access is estimated for the specific health service network associated with Cystic Fibrosis (CF), which is a chronic condition that requires specialty care. Recent studies have shown that Medicaid status is related to survival rate and outcomes [29], but spatial access may also be a factor. The condition has prevalence in the United States of about 30,000 patients with 208 CF care centers in the continental US [30]. Though it is a rare disease, the service network displays heterogeneity, with the spatial access varying greatly over the network.

Focusing on potential spatial access, locations of CF patients are simulated according to the incidence of the disease rather than using existing locations of actual patients (which may be biased by service locations). With CF, the population eligible for Medicaid is considered separately, since they may need to receive service in their home state. 30,000 virtual patients are generated with CF located in county centroids in the continental US, where the prevalence was generated proportionally to the populations in each race/ethnicity who are above or below 2 times the federal poverty level [31], using the incidence matrix for race/ethnicity in Additional file 1 section 5 (see Additional file 5 for raw population data). Patient demand is defined as 10 visits per year to a center (this captures more than 90 % of the patients with location information available in the CF Foundation Registry data) [30]. We assume the actual number of visits is decreasing with the distance to selected service facility, patients will not visit facilities more than 150 miles away (again, this captures more than 90 % of the patients in the registry with location information) [30], and low-income patients will only visit a CF center within the patient’s state due to restrictions of the Medicaid program.

The zip code of each CF center (see Additional file 6) is obtained using patient encounter data from the CF Foundation [30], and the road distance from each CF virtual patient to each CF center is computed using Radical Tools [32] . We assume all facilities are the same size (e.g., can serve 1500 visits a year); the exact number can be changed and the relative comparisons between methods will hold.

Accessibility measures were calculated for E2FSCA, M2SFCA, and the decentralized (with user choice) optimization model. The optimization model was implemented using C++ and the CPLEX solver on a UNIX system (see Additional file 2). The decay functions are such that 10 visits will be made when distance is zero, and visits approach zero when distance is 150 miles; see specific functions in section 7 in Additional file 1: Table S4. There are many functions that can be used to model the decaying willingness of travel. We have chosen to use the exponential function for the rare disease setting of Cystic Fibrosis. Because CF is rare and access to care is relatively low compared to primary care, patients are willing to travel longer distances than for some conditions. The parameter used in the case study was calibrated to be in line with realized utilization derived from the CF registry data (see section 7 in Additional file 1: Figure S12). For the optimization model, a congestion weight of 10 is used unless otherwise specified (see Additional file 1 section 1). For the 2SFCA methods, Medicaid patients were only included in catchment areas of facilities in their own states.

**Fig. 4:**
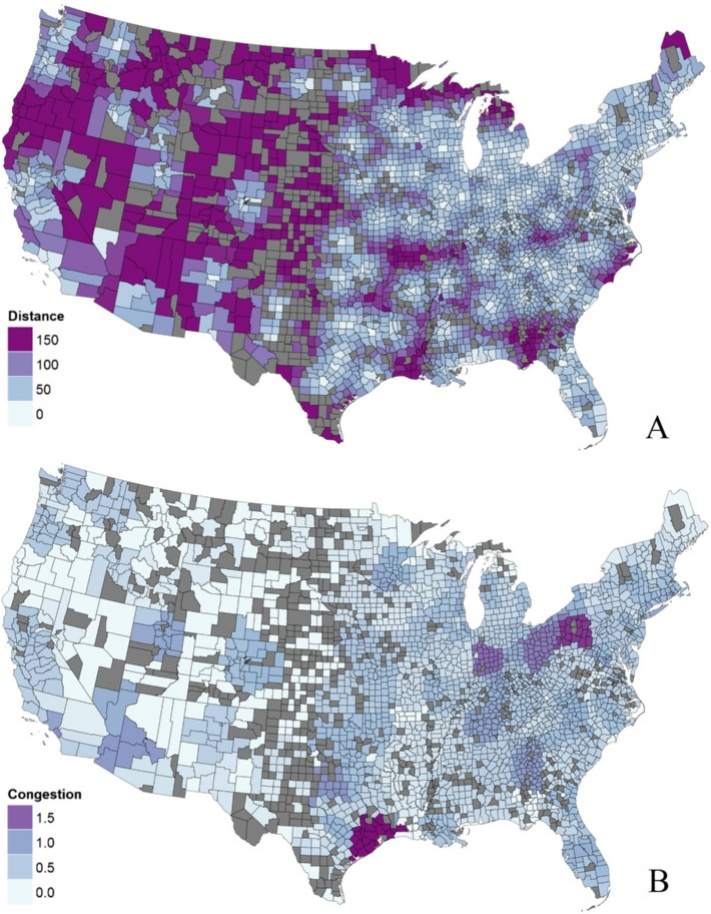
Optimization results for patient cost of potential access. (**a**) Distance, and (**b**) Congestion

Maps of the decentralized optimization model display the distance traveled and the congestion experienced by each person, averaged at the county level, in Fig. 4(a) and 4(b). In general, distance is small close to centers, especially in areas with multiple centers such as the coastal northeast. There are a few pockets with higher distance, especially in parts of the West. Congestion is higher in a few areas, such as around Houston and some parts of Ohio and Pennsylvania. Some counties have no simulated patients, while others have uncovered demand, such as in many counties in the Midwest or Western regions. There are also isolated areas that are uncovered, such as near southwest Georgia, southern Missouri, and some counties at the boundary of the US. A summary histogram is provided for distance, congestion and coverage for each county in Additional file 1 section 6. The distribution of coverage shows that many needed visits are not met, due to the distance patients need to travel to CF centers.

**Fig. 5:**
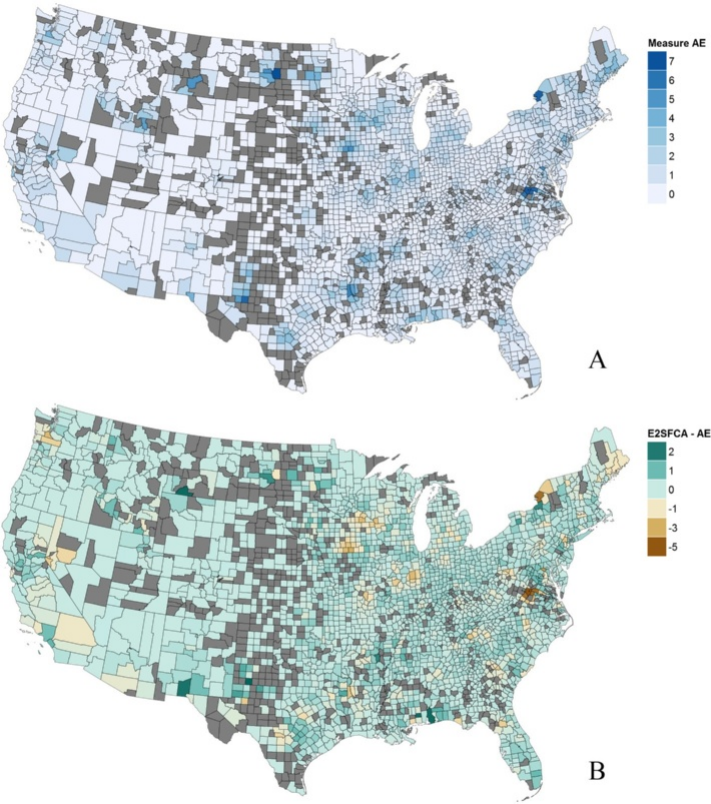
Results comparing optimization model with E2SFCA and M2SFCA for CF care in US. (**a**) Decentralized model composite measure *A* ^*E*^, and (**b**) E2SFCA-*A* ^*E*^

The composite measure *A*^*E*^ generated from the decentralized optimization model is shown in Fig. 5(a). The main areas with high accessibility are near CF centers and around urban areas. There are pockets of low accessibility in many places; however, these can occur for different reasons. In Pittsburg, Pennsylvania, and Columbus, Ohio, Fig. 5(a) shows that the congestion was high, while in Springfield, Missouri, Fig. 5(a) shows that the travel distance is high. Pockets of low accessibility in New York arise from a combination of longer distances and higher congestion.

Figure 5(b) shows the difference between the decentralized optimization model composite measure *A*^*E*^ and the result from the E2SFCA method using the same scale. In comparison to the optimization approach, the E2SFCA method tends to show higher accessibility in areas with many centers (e.g., near Los Angeles and around New York). It also shows higher accessibility in many areas that lie in overlapping service areas for centers (e.g., northern South Carolina, eastern Arkansas, and New Mexico). A pairwise *t*-test (1-tail) shows that for counties with more than 50 CF patients (127 “large” counties) or less than 5 CF patients (1289 “small” counties), the measure from the E2SFCA method is significantly higher than measures from the optimization method (respectively, with p-values 0.20 × 10^−6^ and 2.00 × 10^−2^); for counties of other sizes (“medium” counties), the test is inconclusive. The *F*-test shows that for all groups of counties, the variance of the E2SFCA measure is higher (with *p*-value 1.88 × 10^−4^ for small counties, value less than 10^−6^ for medium counties, and 3.90 × 10^−2^ for large counties. The Mann–Whitney-Wilcoxon test shows that the E2SFCA measure is greater in median than the optimization composite measure with *p*-values less than 10^−6^ for small and medium counties, and 2.02 × 10^−2^ for large counties. The finding is consistent with the analytical results in Additional file 1 section 4 showing that with overlapping catchment areas, E2SFCA quantifies higher access when distances are relatively small. The comparison between the composite measure *A*^*M*^ and the M2SFCA method is similar but the magnitude of differences is smaller.

**Fig. 6:**
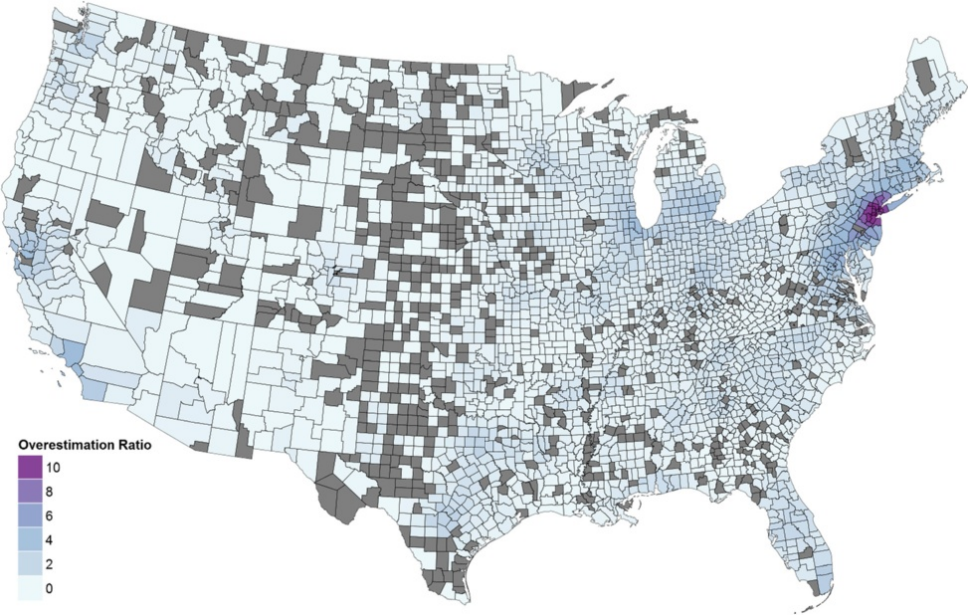
Estimated patient visits in E2SFCA and M2SFCA relative to the visits needed in each county. A value greater than 1 indicates that the 2SFCA methods estimate more visits than needed

The number of visits captured in the E2SFCA method is shown in Fig. 6 in comparison to the visits needed by the population. It is highest around facilities, and especially with multiple facilities such as around New York. For the optimization model, the realized visits per facility are estimated to be 0 to 3000. In contrast, the range for the E2SFCA result is 0 to 10,540 per facility. This is consistent with the analytical result that the number of visits is higher in the E2SFCA approach. The F test indicates that the variance of the facility congestion is significantly higher for the E2SFCA approach, with a *p*-value less than 10^−6^. This is similar to the analytical result that the optimization model always has a lower facility congestion.

**Fig. 7:**
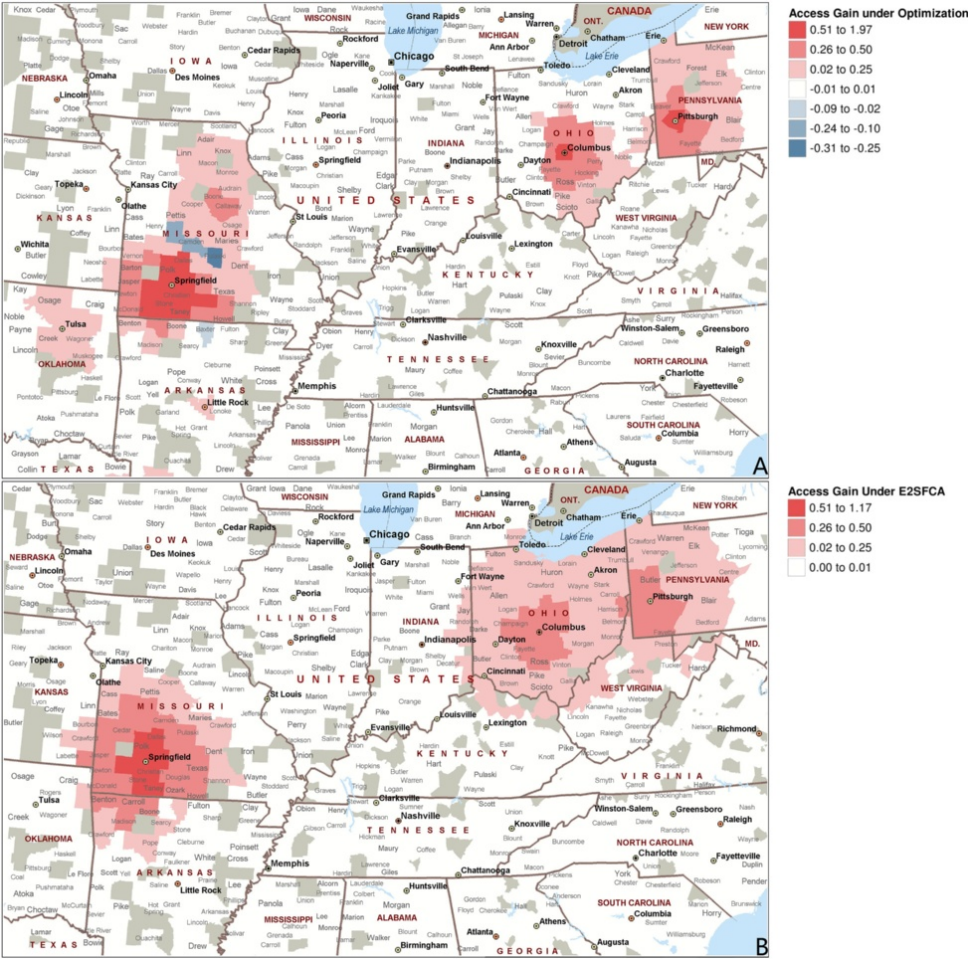
Optimization results showing impact of intervention near locations Springfield, MO, Columbus, OH, and Pittsburgh, PA. (**a**) Access gain under optimization using composite measure *A* ^*E*^, and (**b**) access gain under E2SFCA

The results showing access over the network indicate a number of areas that have uncovered populations, high congestion, and/or high travel distances. Figure 7 shows the results in several local areas after network interventions. One new facility was added to the network in locations with uncovered populations (Springfield, MO), and the capacity of existing facilities was doubled in two locations (Columbus, OH; and Pittsburgh, PA). For the E2SFCA method, the gain in access is centered over the interventions and decays with distance within 150 miles. The gain is positive in all areas with change, as the new facilities increase the opportunities available or have no impact. Under the optimization method, the coverage in an area increases when a new facility is added, and congestion in an area decreases when new capacity is added. Although the total access increases, some populations show a worse composite measure, which indicates that they are traveling shorter distances but experiencing higher congestion (or the reverse) based on new network dynamics. Note also that when the new location is added in Springfield, there are cascading effects under the optimization approach, and access increases for the population around Jefferson City, since their congestion is decreasing due to the new facility. We performed a pairwise *T*-test comparing the impact of intervention on both measures for each of the 479 counties that had a change under the intervention. The test shows that the E2SFCA measure estimates a greater improvement from the intervention compared to the optimization measure, which is consistent with our discussion above.

## Discussion and conclusions

The optimization methods provide several innovations useful both for understanding access and designing interventions. They can be applied across heterogeneous networks with both dense and sparse areas, and they allow user choice to balance travel and congestion within communities. The approach presented includes a way to select the specific parameters of a model. Optimization models also provide both a picture of the status quo and an approach for evaluating a potential change to a network. Fundamentally, the optimization models have a different framework than many catchment methods, since they estimate the access costs associated with a patient’s experience (albeit the *potential* experience rather than actual utilization).

Under optimization models, the presence of additional opportunities only provides gains in potential access when they provide better access compared to existing opportunities, while in the 2SFCA methods, additional opportunities always provides gains in potential access. This difference shows that many 2SFCA methods over count visits when there are facilities with overlapping catchment zones. This effect is stronger in areas with the greatest infrastructure of health services, so interpreting accessibility over a network with sparse and dense areas may not be reasonable. One could adapt the approach by dividing the population by the number of facilities in the zone, or use other adaptations as in the assignment mechanism of the 3SFCA approach. However, these adaptations do not address other issues, such as the cascading effects across the system. The catchment methods tend to capture effects in a defined area, but they do not capture the interactions between areas (or the cascading effects over a network if there are changes introduced) as well as assignment models do. This also means catchment methods may misestimate availability across a network for complicated networks.

Using optimization models for estimating access has many other advantages, as they can be easily adapted to measure access for different types of patients, over different provider types, or with capacity constraints in the network. Moreover, decentralized optimization models allow differences in access in rural and urban areas, which arises directly from the trade-off between distance and congestion rather than solely from different distance functions. It is also easy to modify optimization models to determine the best locations for facilities given an existing demand and supply network [11, 12]. The individual measures from optimization models show not only where to intervene, but also points to what kind of intervention is needed (e.g., new location to reduce distance versus more capacity to reduce congestion). This is especially true as one moves beyond just one measure of access like spatial accessibility to consider the other dimensions of access [1].

This study focus on estimating potential health access using optimization models. There are limitations with the approach. The optimization models assume that patients are trading off travel distance and congestion rationally across a network, while in reality there might be many other factors considered by patients. In addition, the optimization models are built using deterministic known data. In the case study, the possibility of using satellite clinics or services provided through telemedicine are not considered. Results are also dependent on the specific decay function and parameter chosen [27]. Furthermore, the case study also assumes that the transportation modes used by all patients are the same.

Optimization models come at a cost. They are less familiar to many working in public health or public policy. They can be complex to model or compute, although this may be a matter more of the appropriate training than extensive computing power. Sample code and instructions for building the optimization models in this paper are provided for use without a license in Additional file 4 (see Additional file 3 for the software package). It may be most important to use optimization models when a network has facilities with overlapping zones, when one wants to capture the nuances of access across populations, or when one needs to develop interventions to improve access. We hope that the use of optimization models will provoke more discussion in how to measure access, and ultimately how to improve access, especially in light of the increase in computing power and big data that will be coming online in the US health system.

### Availability of supporting data

The data sets supporting the results of this article are included within the article and its additional files (see Additional file 6).

## Additional files

Additional file 1:
**Appendix file that provides detailed supplementary arguments and analysis of the methods and data in the article. **The file also contains a histogram of distances traveled by CF patients compared to estimated distances by the model. Additional file 2:
**Computer codes used to implement the optimization method in this article.**
Additional file 3:
**The free AMPL package that researchers can use to implement optimization models to measure access described in this article.** We provide example codes and instructions to use the AMPL package for researchers who want to implement optimization models to measure access. Additional file 4:
**Contains example codes and instructions to implement optimization models in AMPL and to utilize the free online NEOS solver to solve the optimization models.**
Additional file 5:
**Contains population information to calculate the incidence rate by race/ethnicity who are above or below 2 times the federal poverty level.**
Additional file 6:
**Provides Cystic Fibrosis care center locations and simulated patient locations and demands that this article used to carry out the case study.**

## Abbreviations

2SFCA: Two-step floating catchment area
E2SFCA: Enhanced two-step floating catchment area
M2SFCA: Modified two-step floating catchment area
CF: Cystic fibrosis
