# Supplementary material for: An optimization framework for measuring spatial access over healthcare networks
Source: BMC Health Serv Res. 2015 Jul 17;15:273. doi: 10.1186/s12913-015-0919-8 (PMC4504403; doi:10.1186/s12913-015-0919-8)
Supplement: Additional file 1: — Appendix file that provides detailed supplementary arguments and analysis of the methods and data in the article. The file also contains a histogram of distances traveled by CF patients compared to estimated distances by the model. [file 12913_2015_919_MOESM1_ESM.pdf]

TECHNICAL APPENDIX

The computational codes for the optimization models are available by request from the authors.

1 Parameter Selection and User Choice

In the optimization models, the congestion weight represents the trade-off between willingness to travel and willingness to wait, and it may be adapted to different applications. To identify a range of values that is reasonable for a given problem setting, we quantify several measures of model performance across the network. Below we describe such performance measures, provide the associated principles if applicable, and give calculation details for the CF case.

- Congestion Difference for Close Facilities: *Congestion at facilities that are very close to each other should be similar.* We quantify the absolute value of the difference of each pair of facilities within 50 miles of each other, and sum up the differences over the network.
- Distance Difference (or Congestion Difference) for Close Patients: *Cost experienced by patients who are very close to each other should be similar.* We calculate the variance in distance or congestion across individual visits originating in the same county and sum the values over the network.
- Variance in Distance (or Congestion) across Network: *Heterogeneous networks usually have some disparities in costs experienced; however, very extreme values may not be reasonable.* We quantify the mean distance traveled (or congestion experienced) for visits within a county, then we calculate the variance across the counties in the network.
- Distance Greater than Shortest Distance: *Distance traveled by patients should not be much greater than their shortest possible distances.* Distance to closest facility is compared to average distance traveled by each patient.
- Total Distance or Total Congestion: Calculated across a network by summing up the distance traveled or congestion experienced for each visit to a facility. These two measures are inversely related.

Figure 8 shows the measures for the optimization models under different congestion weights, where the values are normalized [0,1] across results from both models. For patients whose visits are uncovered, we do not include those visits in the calculation of distance or congestion. Note that when the congestion factor is 0, the decentralized optimization is equivalent to the centralized optimization. In this case, the centralized optimization assignment reduces to finding the shortest distance between patients and hospitals. The far right corresponds to splitting congestion evenly among facilities. Thus, the total distance traveled increases with the congestion factor (although not by much), and the total congestion decreases significantly with the congestion factor.

The figure also shows that as the congestion weight increases, the variance of congestion across the network is decreasing, while the variance of distance across the network is

increasing. For a very small congestion factor, distance is very important in the assignment to facilities, and thus facilities that are close to each other may have different levels of congestion. Using the principles above, there should be some differences in congestion and distance across the network, but not excessively large gaps, so we view congestion factors of around 10 as the most reasonable for this setting. The results for the centralized model with different congestion factors are also similar.

Figure 8: *Overall performance measures for different parameter settings of congestion for the decentralized optimization method for Cystic Fibrosis, with highlighted area of recommended values*

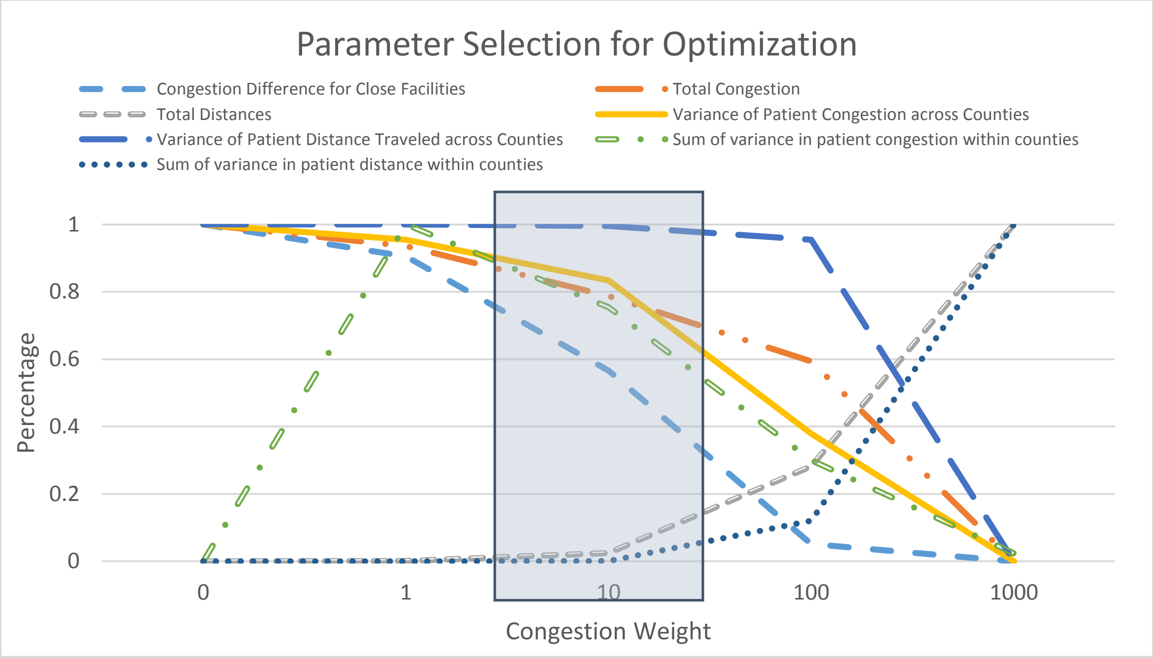

2 Other Variations on Optimization Model

Capacity: Some providers or facilities may have limited resources. This can be introduced by adding a capacity constraint to the basic model. Define c_j = capacity for provider j. The corresponding constraint is

$$\sum_{i=1}^{n} x_{ij} \leq c_j, \forall j = 1, \ldots, m. \quad [8]$$

Unmet Demand: If resources in the network are limited, it may not be possible to meet all demand. In this case, the assignment constraint should be modified to $\leq$. In addition, to ensure that as much demand is met as possible, one can add constraints to ensure that for community i, the minimum service level requirement s_i is met, that is,

$\sum_{j=1}^{m} x_{ij} \geq s_i v_i, \forall i = 1, \ldots, n.$ [9]

Alternatively, one can add a penalty to the objective function for all visits not assigned.

Willingness to Travel: If patients are located too far from providers, they may not be as willing to travel to that provider. In the basic optimization model, the cost to travel is linear with distance. By adjusting the distance values, one can make the cost to travel nonlinear with distance, which represents a patient's higher willingness to travel to close distances. Particular adjustments can be chosen to match the weights of zones as used in the catchment models.

Patient or Provider Types: Some providers choose not to accept Medicaid patients (or limit how many they will accept), which can reduce the spatial access for those patients. One way to represent this in the model is by creating separate assignment variables for each patient type, and adding constraints to limit their assignment to providers with those preferences (33).This allows the optimization approach to incorporate the link between affordability and spatial accessibility.

On the demand side, patients may have preferences for providers with certain characteristics, e.g., children and their caregivers may desire providers focused on pediatric care. One way to incorporate this is to adjust the travel cost to be relatively lower for providers of the preferred characteristics. This example shows how the optimization model can incorporate *acceptability* (1) in the measurement of access. A similar approach (adjusting distances) can be used to capture differences in patient mobility, e.g., for families with automotive vehicles or not.

Objective Function: In Section 2.1 we describe a model with an objective function that has a particular congestion cost. Many other variations on congestion are possible, including linear with the number of visits at a facility, exponential with the number of visits, or others. More generally, many variations on the objective function are possible. *Interventions*: Decision variables can be added to optimization models to represent whether or not a new facility should be located in a network at particular locations, whether or by how much to increase capacity, or other interventions. The interventions can be designed to optimize the overall system performance or to reduce the disparities among subpopulations.

3 Minimum cost network flow transformation for decentralized model

Decision Variables:
x_{ij} = the percentage of time that patients in location i visit facility j
$y_{jk} = 1$ if the kth visit is selected for facility j
Parameters:
d_{ij} = distance between patient location i and facility j
$w(d_{ij})$ = decay function value for distance d_{ij}
v_i = demand of patient location i

C_j = capacity at facility j
$f_k = k$, the cost of marginal congestion for the kth visit
α = congestion weight
Model:

$$\min Z = \sum_{i=1}^{n}\sum_{j=1}^{m}\frac{d_{ij}}{w(d_{ij})}x_{ij}v_i w(d_{ij}) + \alpha\sum_{j=1}^{m}\frac{1}{C_j}\sum_{k=1}^{n}f_k y_{jk}$$

Constraints:

$\sum_{j=1}^{m} x_{ij}v_i = v_i, \forall i = 1, \dots, n$ (assignment constraint)

$\sum_{i=1}^{n} x_{ij}v_i w(d_{ij}) = \sum_{k=1}^{n} y_{jk}, \forall j = 1, \dots, m$ (flow balance constraint)

$x_{ij} \geq 0, \forall i = 1, \dots, n$ and $\forall j = 1, \dots, m.$
$0 \leq y_{jk} \leq 1, \forall i = 1, \dots, n$ and $\forall j = 1, \dots, m.$

4 Analytical Results: Networks with Overlapping Service Areas

Result 4: Optimization models show higher accessibility in non-overlapping service areas.

It can be difficult to understand model differences across complex networks like we study in Section 4. Thus we analyze one more simulated system that can assist in making comparisons between the 2SFCA approaches and optimization models.

1. When the population density is homogenous over the network:

Consider System I with two facilities, each with a population surrounding them in a circle of radius R. The distance between the two facilities is also R, so some population resides between both facilities. Define the decay function $\boldsymbol{w(d_{ij}) = e^{-d_{ij}}}$, where $\boldsymbol{0 \leq d_{ij} \leq R}$. The density of the areas is 1 unit per square mile and the supply C is the same in each facility. We will compare composite measures across the network in Figure 9.

For 2FSCA the physician-to-population ratio at each facility is $S = \frac{C}{V}$, where $V = \int_0^{2\pi}\int_0^{r} e^{-r} r dr d\theta$ denotes the number of visits. For the population inside the catchment of only one facility, each patient's accessibility can be calculated by $S_{P_s}^{r} = e^{-r}\frac{C}{V}$, where r is the distance between the patient and the facility. For the population in overlapping catchment areas, a patient's accessibility can be calculated as $S_{P_o}^{r_1,r_2} = (e^{-r_1} + e^{-r_2})\frac{C}{V}$. Where r_1 is the distance to the first facility and r_2 is the distance to the second facility. We also have $r_1 + r_2 \leq R, r_1, r_2 \leq R, \Rightarrow 2e^{-R} \leq e^{-r_1} + e^{-r_2} \leq 2e^{-\frac{R}{2}}$.

For the optimization models, we initially use a congestion weight such that patients will visit their closest facility. The congestion at each facility is $F = \frac{\frac{2}{3}V + M}{C}$, where $M =$

$\int_0^{\frac{1}{3}\pi} -\left(\frac{R}{2cos\theta}\right) e^{-\frac{R}{2cos\theta}} d\theta < \frac{1}{3}V$. For the population inside the catchment of only one facility, the patient's congestion is $F_{P_S} = F$. For the population in the overlapping catchment areas the congestion experienced by each patient is $F_{P_O} = F$.

If a patient is inside a single circle, then the optimization model shows higher accessibility than the 2SFCA approaches since $F_{P_S} = F < \frac{1}{S}$. This is true for larger congestion weights if there is no decay function or if the congestion weights are extreme. The result occurs because visits are over-counted in the 2SFCA methods, while the optimization model is capturing the cost associated with user experience. For patients in the overlapping areas, we find that which method estimates higher accessibility depends on the value of radius R. If $R < ln\frac{4}{3}$, then the accessibility for patients in the middle is: $S_{P_O}^r \geq \frac{2e^{-R}C}{V} > \frac{3C}{2V} > \frac{1}{F_{P_O}}$. This implies that the overall range of accessibility in the optimization model is smaller than the 2SFCA methods, so the access appears smoother. R values that are small represent dense areas.

Figure 9: *Systems I and II have populations distributed in circles around facility 1 and 2. In I, the density is 1 person per square mile, and in II the density is 1 and n per square mile for the left and right circles, respectively. In I, the figure indicates the locations of populations M and ϵ used in the calculations.*

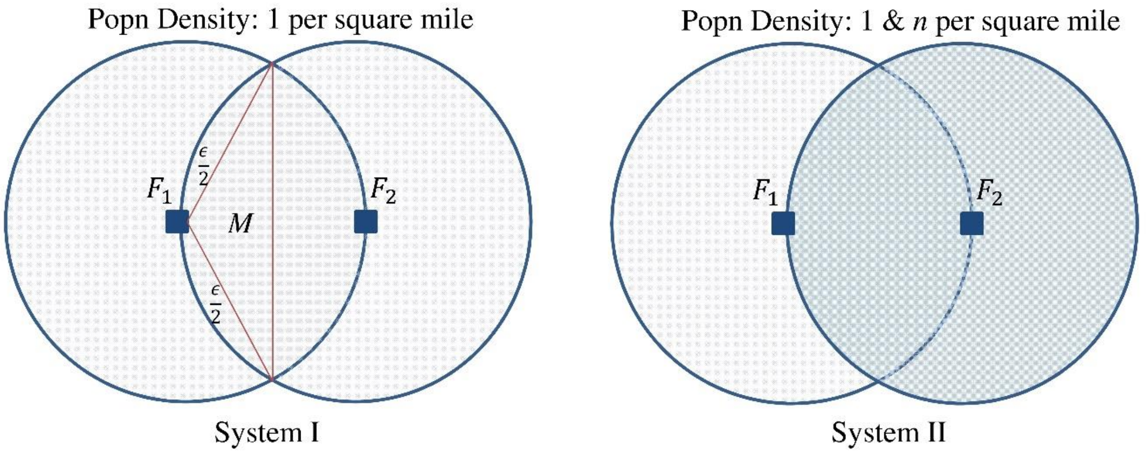

2. When the population density is non-homogenous over the network

Consider system II. The E2SFCA facility and patient level accessibility measures are:

Facility 1: $S_1 = \frac{C}{V+(a-1)(\frac{V}{3}+\epsilon)}, \epsilon = \int_{\frac{\pi}{3}}^{\frac{\pi}{2}} \int_0^{2Rcos\theta} e^{-r} r dr d\theta.$

Facility 2: $S_2 = \frac{C}{aV}, \epsilon = \int_{\frac{\pi}{3}}^{\frac{\pi}{2}} \int_0^{2Rcos\theta} e^{-r} r dr d\theta.$

For a patient inside the catchment of facility 1 only: $S_{P_1}^r = e^{-r} S_1$.

For a patient inside the catchment of facility 2 only: $S_{P_2}^r = e^{-r} S_2$.

For a patient inside the overlapping area: $S_{P_o}^{r_1,r_2} = e^{-r_1}S_1 + e^{-r_2}S_2, r_1 + r_2 \leq R, r_1, r_2 \leq R, \Rightarrow 2e^{-R} \leq e^{-r_1} + e^{-r_2} \leq 2e^{-\frac{R}{2}}$.

The facility and patient level congestion measures for Shortest Distance are:

Facility 1: $F_1 = \frac{\frac{2}{3}V + aM + (a-1)\epsilon}{C}$.

Facility 2: $F_2 = \frac{a(\frac{2}{3}V + M)}{C}$.

For a patient inside the catchment of facility 1 only: $F_{P_1} = F_1$.

For a patient inside the catchment of facility 2 only: $F_{P_2} = F_2$.

For a patient inside the overlapping area: $F_1 \leq F_{P_o} \leq F_2$.

For this system, again we have at the facility level: $F_1 < \frac{1}{S_1}, F_2 < \frac{1}{S_2}$.

At the patient level, it is obvious that $S_{P_1}^r < F_{P_1}$ and $S_{P_2}^r < F_{P_2}$.

If $R < \ln(\frac{2}{3} + \frac{2M+\epsilon}{V})$, then the access under Shortest Distance has a smaller range (i.e., is “smoother”). (This R is guaranteed to exist since $\epsilon' < \epsilon, M' < M \Rightarrow \frac{1}{3}V < 2M + \epsilon \Rightarrow \frac{2}{3} + \frac{2M+\epsilon}{V} > 1$.)

5 Incidence matrix for race/ethnicity

Table 3: *Incidence rate of Cystic Fibrosis by race/ethnicity (34, 35)*

Race/Ethnicity	Incidence
White (non-Hispanic)	1/3000
Hispanic White	1/13500
African American	1/15000
Asian	1/30000

6 Additional Figures for Case Study

Figure 10: *Distribution of simulated population of CF patients in US where uncolored counties have no patients (A); and locations of CF centers (B).*

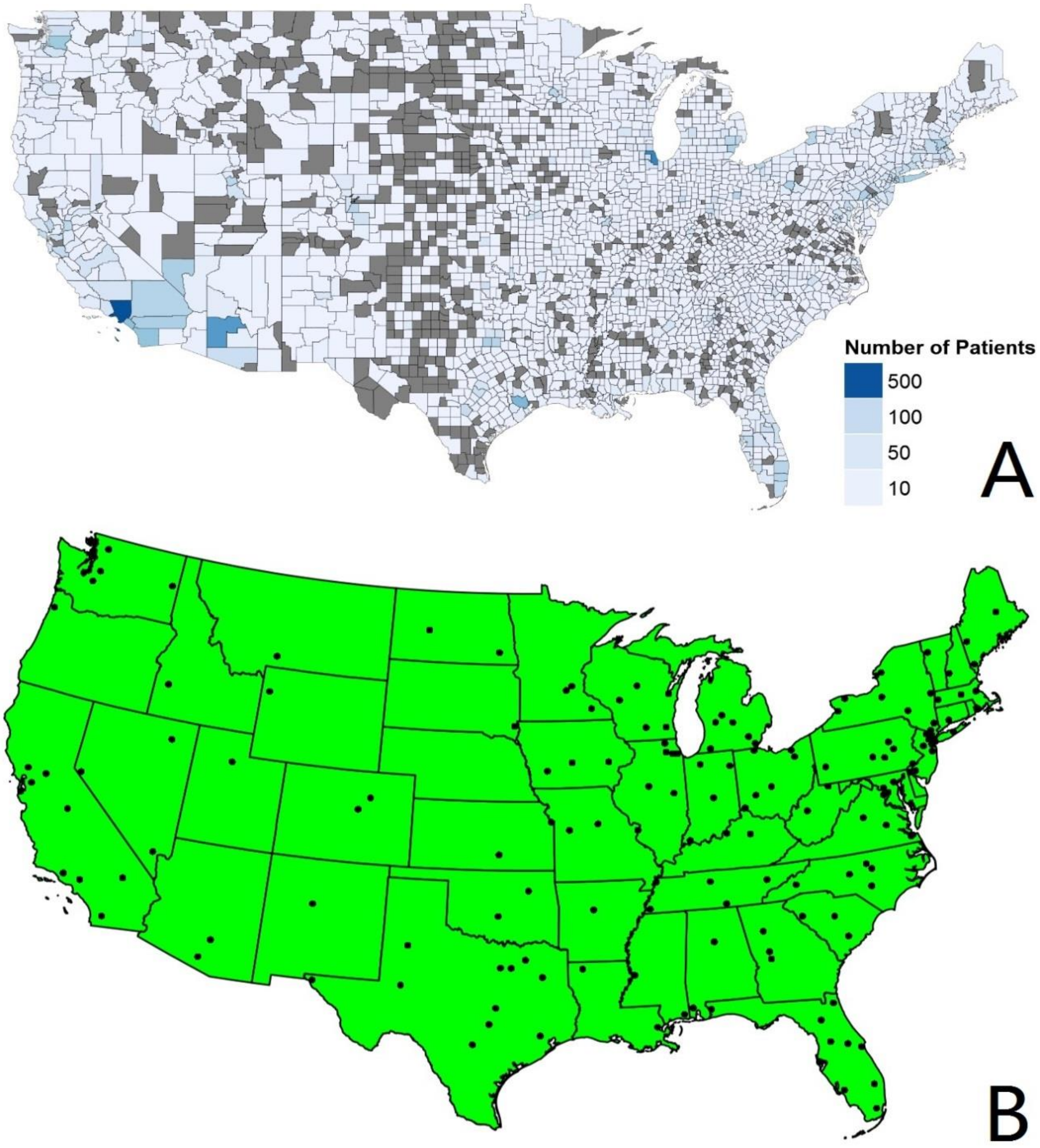

Figure 11: *Histograms of optimization model results. (A) Congestion, (B) distance, and (C) coverage. (3-column fitting image)*

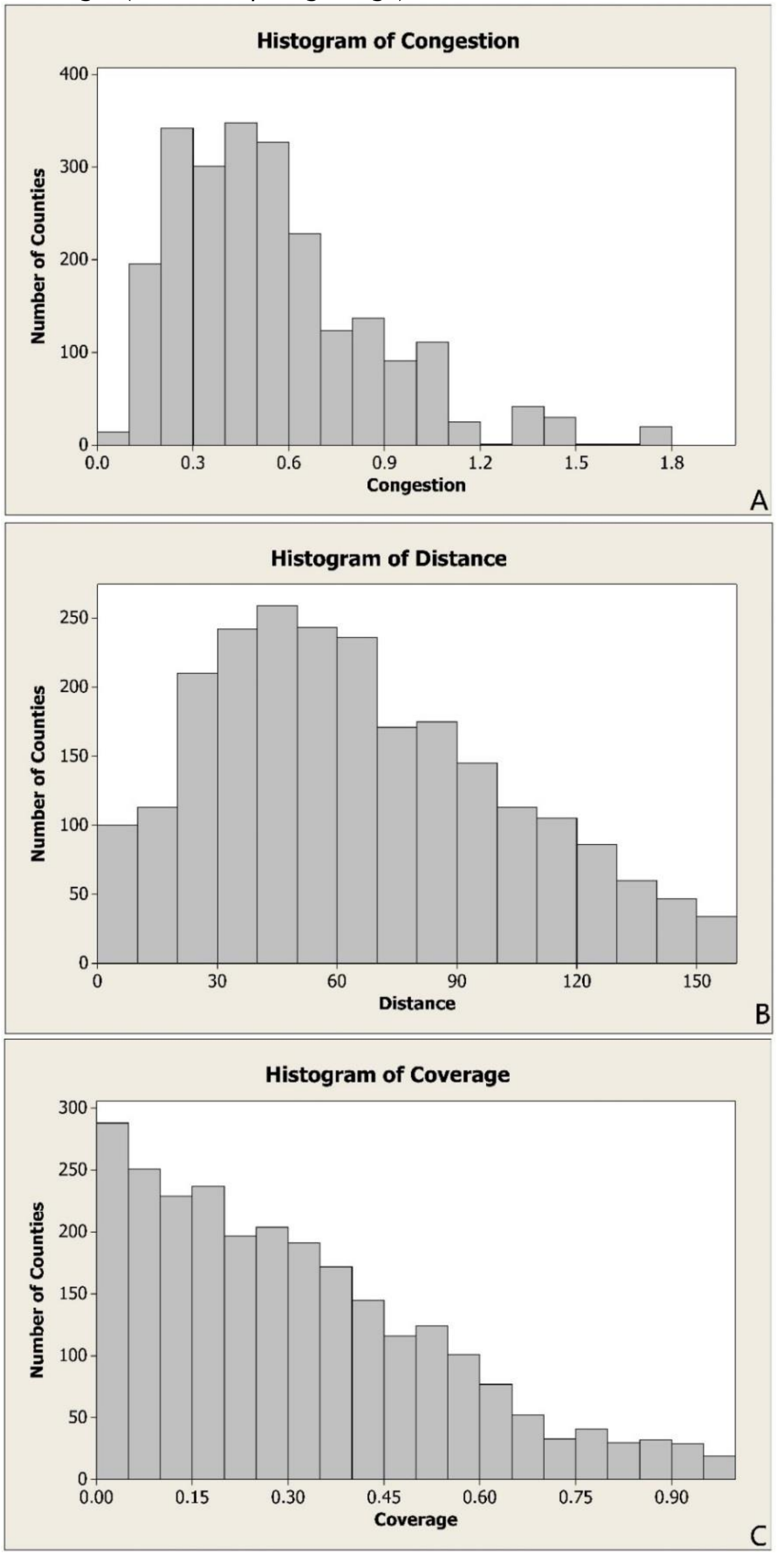

7 Model specifics: Cystic Fibrosis Implementation

Figure 12: *Figure displays the percentage visits to Cystic Fibrosis care centers from 1997 to 2013 of different distances along with the visits quantified by an exponential decay function with parameter = 0.02.*

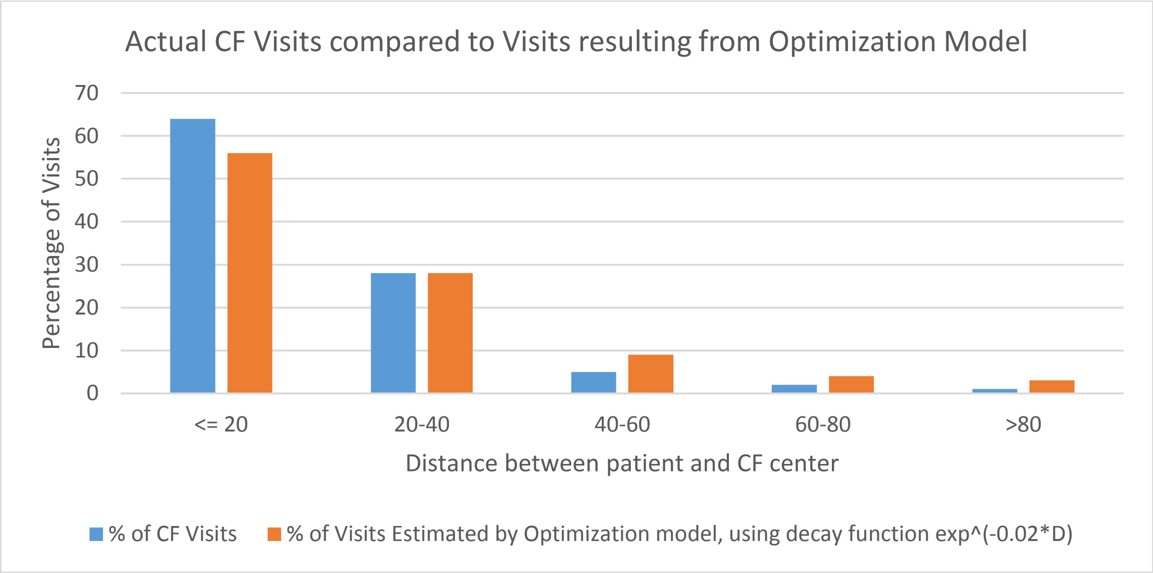

Table 4: *Specific values for each method in the CF case study.*

	2SFCA Methods		Optimization Model
Zone 1	$w_1 = e^{-0.02*\frac{0+50}{2}} = 0.6065$		$d_{ij}^{adj} = d_{ij}e^{0.02*d_{ij}}$, $\forall d_{ij} \leq 150$ miles, $d_{ij}^{adj} = 9999$, $\forall d_{ij} > 150$ miles.
Zone 2	$w_2 = e^{-0.02*(50+100)/2} = 0.2231$		
Zone 3	$w_3 = e^{-0.02*(100+150)/2} = 0.0821.$		
# Communities with CF patients	2568		
Number of Facilities	208		209 (1 dummy location for uncovered demand)
Visit Capacity per Facility	1500		

We estimate the number of visits for each patient-CF center pair *i,j* based on an exponentially decaying function $v_{ij} = 10e^{-0.02d_{ij}}, d_{ij} \leq 150$ miles, and $v_{ij} = 0\ , d_{ij} >$ 150 miles. For all patients with family income below two times the FPL, $v_{ij} = 0$ for all centers located in other states.

For the 2SFCA methods, the three catchment zones are defined by 0-50, 50-100, and 100-150 miles. We set $v_i = 10$, $c_j = 1500$ for $j = 1, \ldots, m$, and we quantify w_i on zone $i, i = 1,2,3$ as

$$w_1 = e^{-0.02*\frac{0+50}{2}} = 0.6065$$

$$w_2 = e^{-0.02*(50+100)/2} = 0.2231$$

$$w_3 = e^{-0.02*(100+150)/2} = 0.0821$$
