# Supplementary material for: An optimization framework for measuring spatial access over healthcare networks
Source: BMC Health Serv Res. 2015 Jul 17;15:273. doi: 10.1186/s12913-015-0919-8 (PMC4504403; doi:10.1186/s12913-015-0919-8)
Supplement: Additional file 4: — Contains example codes and instructions to implement optimization models in AMPL and to utilize the free online NEOS solver to solve the optimization models. [file 12913_2015_919_MOESM4_ESM.zip › AMPL Instruction.doc]

The AMPL Package can be downloaded from the website: http://www.ampl.com/DOWNLOADS/

.mod file:

The .mod file contains a generic formulation of the decentralized optimization model similar to what we implemented in our paper. The corresponding mathematical formulation can be founded in the Technical Appendix (of the paper). This basic formulation only contains one type of patient and facility (i.e., only one set of distance matrix), and we assume the decay function is 1 for all distance (for other decay functions, please refer to the comments inside the .mod file). Users are welcome to modify the basic formulation we provide tailor to their specific needs.

.dat file:

The data file contains parameter values specific to applications. In this file we specify the distance matrix, capacity (or size of facility) vector, demand vector, (starting) congestion weight and possibly the decay function. For users who want to use the basic formulation, it is sufficient to change the .dat file only.

.run file:

The .run file contains a list of commands that allows users to run multiple iterations and display all decision variable values. In our example, we run congestion weight from 1 to 10 and display the Assign and Congestion decision variables as matrices for each iteration. Users can further calculate the congestion experienced by each individual by first computing the facility level congestion by summing up each column of Congestion for each facility, and then taking weighted average over facility congestions based on the Assign matrix for each patient or community.

After creating the .mod, .dat, and .run files, go to <http://neos.mcs.anl.gov/neos/solvers/> to submit jobs. From the list of solvers, click one with AMPL input. For example, we demonstrate how to use Gurobi to solve a linear programming problem: go to <http://neos.mcs.anl.gov/neos/solvers/lp:Gurobi/AMPL.html>; upload the .mod, .dat and .run files to the respective places; add your email address to receive the results and hit Submit to NEOS to submit job. The output file will be emailed to users. (For centralized optimization, use MINOS)
